# Chronic Glutathione Depletion Confers Protection against Alcohol-induced Steatosis: Implication for Redox Activation of AMP-activated Protein Kinase Pathway

**DOI:** 10.1038/srep29743

**Published:** 2016-07-12

**Authors:** Ying Chen, Surendra Singh, Akiko Matsumoto, Soumen K. Manna, Mohamed A. Abdelmegeed, Srujana Golla, Robert C. Murphy, Hongbin Dong, Byoung-Joon Song, Frank J. Gonzalez, David C. Thompson, Vasilis Vasiliou

**Affiliations:** 1 Department of Environmental Health Sciences, Yale University, New Haven, CT 06520, USA.; 2 Department of Social Medicine, Saga University School of Medicine, Saga, 849-8501, Japan; 3 Laboratory of Metabolism, Center for Cancer Research, National Cancer Institute, Bethesda, Maryland 20852, USA; 4 Laboratory of Membrane Biochemistry and Biophysics, National Institute on Alcohol Abuse and Alcoholism, National Institutes of Health, Bethesda, MD 20892, USA; 5 Department of Pharmacology, University of Colorado AMC, Aurora, CO 80045, USA; 6 Department of Clinical Pharmacy, University of Colorado AMC, Aurora, CO 80045, USA.

## Abstract

The pathogenesis of alcoholic liver disease (ALD) is not well established. However, oxidative stress and associated decreases in levels of glutathione (GSH) are known to play a central role in ALD. The present study examines the effect of GSH deficiency on alcohol-induced liver steatosis in *Gclm* knockout (KO) mice that constitutively have ≈15% normal hepatic levels of GSH. Following chronic (6 week) feeding with an ethanol-containing liquid diet, the *Gclm* KO mice were unexpectedly found to be protected against steatosis despite showing increased oxidative stress (as reflected in elevated levels of CYP2E1 and protein carbonyls). *Gclm* KO mice also exhibit constitutive activation of liver AMP-activated protein kinase (AMPK) pathway and nuclear factor-erythroid 2–related factor 2 target genes, and show enhanced ethanol clearance, altered hepatic lipid profiles in favor of increased levels of polyunsaturated fatty acids and concordant changes in expression of genes associated with lipogenesis and fatty acid oxidation. In summary, our data implicate a novel mechanism protecting against liver steatosis *via* an oxidative stress adaptive response that activates the AMPK pathway. We propose redox activation of the AMPK may represent a new therapeutic strategy for preventing ALD.

Alcoholic liver disease (ALD) is a major cause of chronic liver disease, and accounts for over 30,000 deaths annually in the United States^1^. The majority of ingested ethanol is metabolized in the liver through two sequential steps^2^. Ethanol is oxidized to acetaldehyde by alcohol dehydrogenase I (ADH1), catalase (CAT) and/or cytochrome P450 2E1 (CYP2E1). Acetaldehyde is then oxidized by acetaldehyde dehydrogenases (ALDH2, ALDH1B1 and ALDH1A1) to acetate. During the development of ALD, numerous pathways in the liver are modulated by alcohol, many through mechanisms involving oxidative stress^3^. Ethanol metabolism, CYP2E1 induction, compromised antioxidant defenses, mitochondrial injury, activation of Kupffer and stellate cells, hypoxia, and iron overload can all contribute to the alcohol-induced oxidative environment. Accumulation of ethanol-associated reactive molecules, e.g., reactive oxygen (ROS) and nitrogen species (RNS), and electrophilic products, e.g., acetaldehyde and lipid peroxidation-derived products, can be harmful to biological systems due to their propensity to inactivate enzymes, leading to loss of protein function, DNA damage and cell death^4^. Manifestations of hepatic oxidative damage include dysregulation of lipid metabolism (leading to steatosis), hepatocyte degeneration and death, and activated immune response (leading to inflammation and fibrosis/cirrhosis). Importantly, these are all features of ALD depending on the disease stage^5^.

Glutathione (GSH) is the most abundant cellular non-protein thiol, attaining millimolar concentrations in the liver^6^. It acts as an antioxidant by directly scavenging free radicals or serving as a cofactor for antioxidant enzymes^6^. Because of its abundance, GSH plays a key role in maintaining cellular redox homeostasis and in cellular mechanisms that protect against oxidative stress. The function of GSH in hepatocytes has been investigated by pharmacological inhibition or genetic ablation of the glutamate-cysteine ligase (GCL), the rate-limiting and regulatory enzyme in GSH biosynthesis. In higher eukaryotes GCL is a heterodimer comprising a catalytic (GCLC) and a modifier (GCLM) subunit^7^. Transgenic mice in which GCLC expression is abolished in hepatocytes have 5–8% of normal hepatic GSH levels and manifest liver pathologies characteristic of the various clinical stages of fatty liver disease^8^,^9^. Treatment of newborn rats with L-buthionine sulfoximine (BSO), an irreversible inhibitor of GCLC, leads to hepatic abnormalities including mitochondrial dysfunction^10^. These studies underscore the essential role of GSH in normal functioning of the liver.

It has been proposed that depletion of hepatic GSH, particularly mitochondrial GSH, is one of the early changes associated with chronic alcohol consumption and is a critical contributor to ALD pathogenesis^11^. Nevertheless, the effect of ethanol consumption on the total hepatic GSH pool is equivocal in that studies have shown decreased, unchanged or increased levels^12^,^13^,^14^, most likely related to differences in ethanol exposure regimes and/or analytical assays. In one study, BSO treatment alleviated ethanol-induced elevations in serum ALT and liver TG content^15^. Given that BSO possesses non-specific pharmacological activities, the authors concluded that this effect was independent of the inhibition of GSH biosynthesis^15^. Collectively, the pathogenic role of GSH depletion in alcohol-induced liver injury remains to be defined.

In the current study, we utilized a transgenic mouse model to elucidate the role of GSH in hepatic responses to chronic ethanol consumption and explored underlying mechanisms. Global disruption of *Gclm* results in mice (GCLM knockout) that have ≈15% of hepatic GSH levels seen in wild-type mice^16^. The 85% depletion of hepatic GSH results in a decreased GSH redox potential (ΔE_GSH_). Mitochondrial GSH is maintained at 40% of the wild-type level and is accompanied by increased H_2_O_2_ release. Nevertheless, *in vitro* mitochondrial function was indistinguishable from wild-type^17^. The viability and good health of naive GCLM knockout mice makes them a valuable model for studying the impact of chronic GSH deficiency^18^. Following 6 wk of ethanol administration, GCLM knockout mice were resistant to alcohol-induced steatosis and exhibited beneficial metabolic and stress responses to chronic ethanol consumption.

## Results

### Protection from alcohol-induced steatosis and accelerated clearance of ethanol and acetaldehyde in KO mice

**Figure 1:**
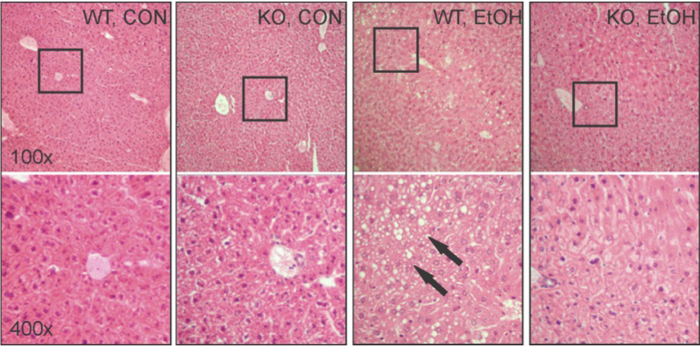
Resistance to ethanol (EtOH)-induced steatosis in GCLM KO mice. Representative images of liver histology by H&E staining. Squares in upper panels were enlarged in the lower panels. Excessive fat accumulation (steatosis) primarily in the form of macrovesicles (*arrows*) was only observed in EtOH-fed WT mice. CON, control diet; EtOH, ethanol diet. Magnifications: 100x (upper panels) and 400x (lower panels).

Mice were fed a high fat liquid diet containing ethanol (EtOH) or isocaloric control (CON) diet for 6 wk. For EtOH-fed mice, the diet contained 2% v/v EtOH (10.8% total calories) in the first wk and the ethanol content increased weekly by 1% until reaching 5% v/v (27% total calorie). Following the 6-wk feeding period, average daily intake (calories) by WT and KO mice was no different (Table 1). Compared with EtOH-fed WT mice, KO mice were less susceptible to EtOH-induced deleterious effects, including body weight loss (reflected by less body weight gain), liver weight gain and hepatocyte damage (reflected by plasma ALT activity) (Table 1). Most interestingly, the steatosis observed in EtOH-fed WT mice was absent from KO mice (Fig. 1). In line with histological observations, EtOH feeding caused a 50% increase in total hepatic TG content in WT mice, but had no effect on the hepatic TG content in KO mice (Table 1). It is worth noting that histological examination of the liver revealed no significant liver inflammation in either WT or KO mice (Fig. 1), which is further supported by unaltered mRNA levels of inflammatory genes TNFα, IL-6 and IL-1β (supplementary Fig. 1).

**Figure 2:**
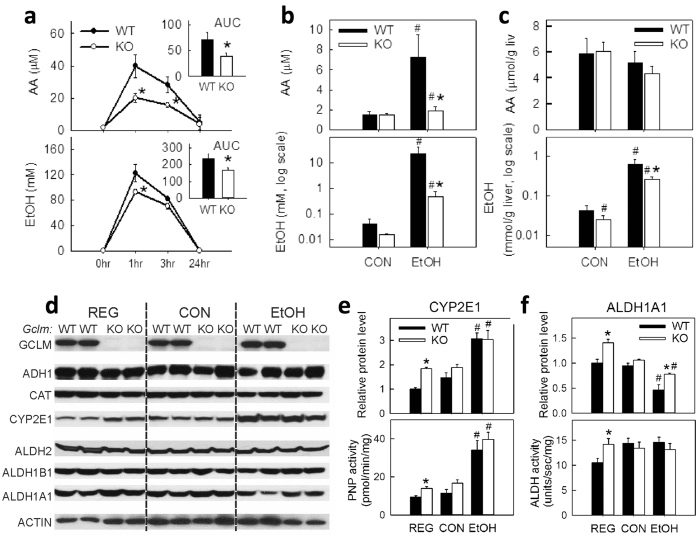
Accelerated clearance of ethanol (EtOH) and acetaldehyde (AA) in GCLM KO mice. (**a**) EtOH and AA pharmacokinetics following acute EtOH administration (5 g/kg, i.p). Inset (*upper right*): area under the curve (AUC). Data represent mean ± SEM from 4–6 mice. *P < 0.05, vs. WT mice. Concentrations of EtOH and AA in blood (**b**) and liver (**c**) from mice fed a control (CON) or EtOH diet for 6 wk. Expression of EtOH- (ADH1, CAT and CYP2E1) and AA- (ALDH2/1A1/1B1) metabolizing enzymes (**d**), protein levels and enzymatic activity of CYP2E1 (**e**) and ALDH1A1 (**f**) in livers from mice fed regular chow (REG), CON or EtOH liquid diets for 6 wk. CYP2E1 activity was expressed as *p*-nitrophenol (PNP) oxidation activity. Data are mean ± SEM from 4 mice. ^*^P < 0.05, vs. diet-matched WT mice. ^#^*P* < 0.05, vs. CON-fed mice of the same genotype.

Pharmacokinetics of blood EtOH and acetaldehyde (AA) in KO mice were compared with those in WT mice (Fig. 2a). Following an acute administration of EtOH (5 g/kg, i.p.), KO animals exhibited 30% and 50% less EtOH and AA accumulation in circulation, respectively, suggesting an increased capacity for liver EtOH and AA metabolism in KO mice. At the end of the 6-wk EtOH feeding, a similar trend was also observed in blood (Fig. 2b) and liver (Fig. 2c). Evaluation of EtOH and AA metabolizing enzymes (Fig. 2d) revealed constitutive induction of CYP2E1 (1.8-fold) and ALDH1A1 (1.4-fold) at both protein and enzymatic activity levels in KO livers relative to WT livers (Fig. 2e,f). However, ethanol feeding induced CYP2E1 expression in WT mice to levels comparable to those observed in KO mice (Fig. 2e).

### Persistent oxidative stress and induction of nuclear-factor-erythroid 2–related-factor 2 (NRF2) antioxidant response in KO livers

**Figure 3:**
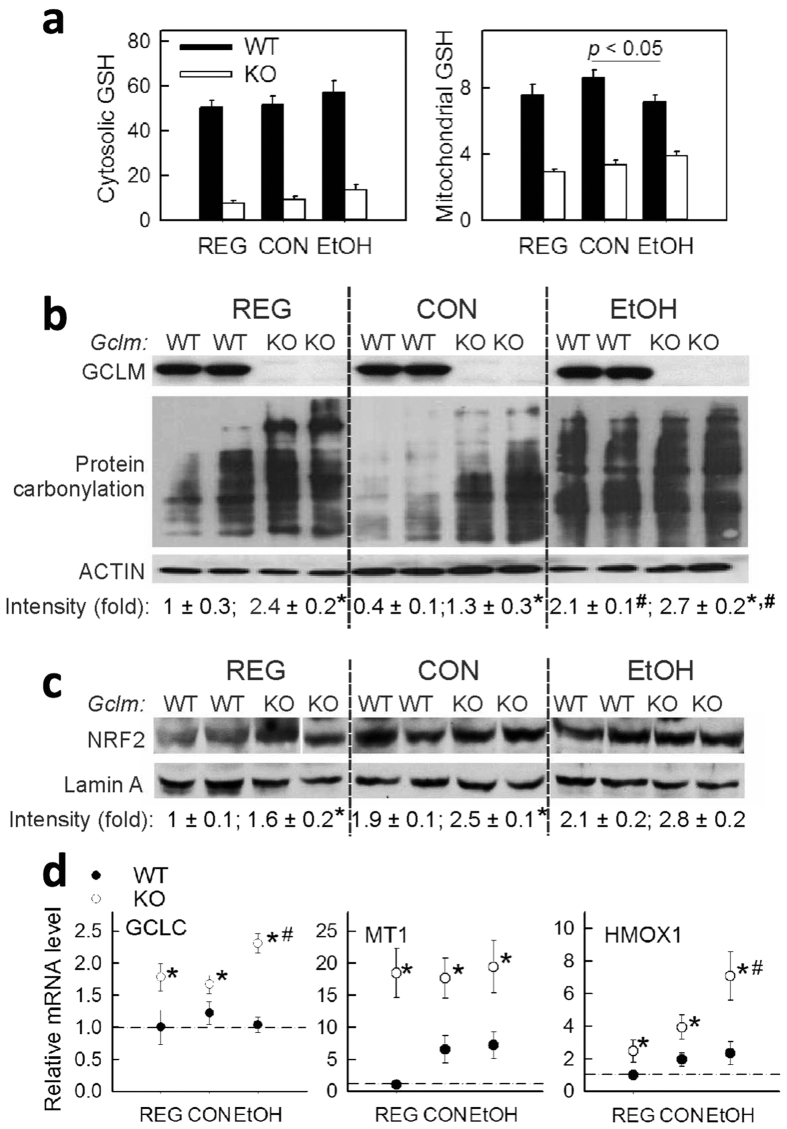
Persistent oxidative stress and induction of NRF2 antioxidant response in GCLM KO livers. (**a**) liver cytosolic and mitochondrial GSH levels (nmol/mg protein) in livers from mice fed regular chow (REG), control (CON) or ethanol (EtOH) liquid diets for 6 wk. Data are mean ± SEM from 5–6 mice. (**b**), levels of carbonylated proteins in liver homogenates. *C*, Nuclear accumulation of NRF2 protein. Protein band intensity was quantified by densitometry and reported as fold of control (REG-fed WT mice) after normalization to β-actin (**b**) or lamin-A (**c**). Data are mean ± SEM from 4 mice. (**d**) Q-PCR analysis of liver mRNA for the oxidative stress responsive genes GCLC, metallothionein-1 (MT1) and heme oxygenase-1 (HMOX1). Relative mRNA levels were expressed as fold of control (REG-fed WT mice; *dashed line*), after normalization to the housekeeping gene β-2 microglobulin (B2M). Data are mean ± SEM from 4–6 mice. ^*^P < 0.05, vs. diet-matched WT mice. ^#^*P* < 0.05, vs. CON-fed mice of the same genotype.

EtOH feeding decreased GSH levels specifically in the mitochondrial pool of livers of WT mice (Fig. 3a). Although KO mice had significantly lower levels of GSH in the cytosol and mitochondria, EtOH did not decrease it further (Fig. 3a). Consistent with ≈85% depletion of hepatic GSH, the KO liver showed much higher basal levels of protein carbonylation (Fig. 3b), suggesting increased oxidative stress. When compared with the respective CON-fed groups, EtOH dramatically induced hepatic protein carbonylation in both WT and KO mice, albeit the levels in the KO mice remained higher than those observed in WT mice. The sustained oxidative stress status in KO livers was paralleled by increased levels of transcription factor NRF2 in the nucleus (Fig. 3c). Transactivation of NRF2 in KO livers was further supported by constitutive induction of NRF2 target genes, including *Gclc*, metallothionein I (*Mt1*) and heme-oxgenase 1 (*Hmox1)* (Fig. 3d). Importantly, expression levels of these genes have been used in numerous studies as sensitive indices for oxidative stress^8^,^19^,^20^.

### Differential changes in lipid profiles from KO livers following chronic alcohol feeding

**Figure 4:**
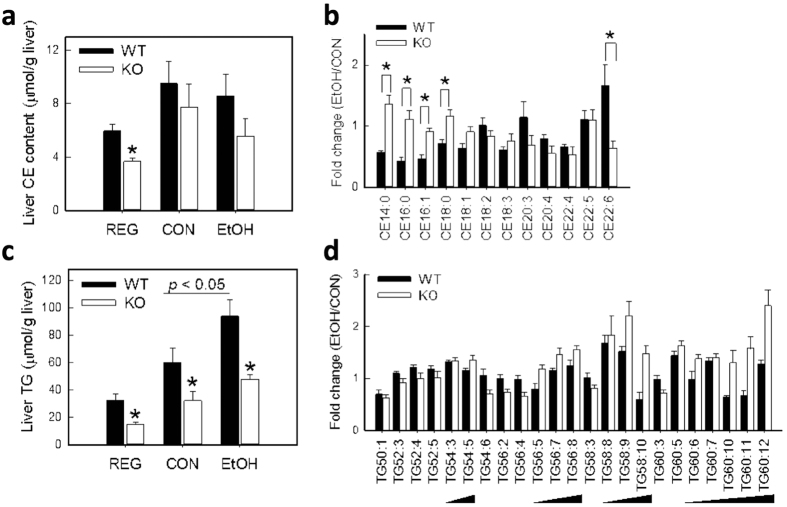
Lipidomic analysis of liver cholesterol ester (CE) and triglycerides (TG). Hepatic neutral lipids were profiled by mass spectrometry from liver lipid extracts obtained from WT and KO mice fed regular chow (REG), control (CON) or ethanol (EtOH) liquid diets for 6 wk. Total liver CE (**a**) and TG (**c**) content were calculated by summing the amounts of all molecular species present in each lipid class. Relative abundance of each lipid species in the CE (**b**) and TG (**d**) class upon EtOH treatment was calculated as the fold change in respective CON-fed groups. Data are mean ± SEM from 4–5 mice. ^*^P < 0.05, vs. diet-matched WT mice.

Histological and biochemical examination of the liver demonstrated KO mice to be resistant to EtOH-induced steatosis (Fig. 1 and Table 1). To characterize the nature of changes in lipid composition by genotype and/or by EtOH treatment, hepatic neutral lipids (including cholesterol ester (CE) and triglyceride (TG)) were quantitated and profiled. Both lipid families showed differences in total hepatic content that were genotype-dependent (Fig. 4a,c), as well as changes in specific lipid species that were influenced by EtOH treatment (Fig. 4b,d). Specifically, total hepatic CE content in KO mice was 40% lower than in WT mice fed regular chow diet; such a difference was not observed following liquid diet treatment (Fig. 4a). While EtOH did not affect the total content of CE regardless of the genotype (Fig. 4a), saturated and monounsaturated CE species were maintained at higher levels in the KO liver relative to the WT liver following chronic EtOH consumption (Fig. 4b). On the other hand, EtOH feeding increased hepatic TG concentration by 50% in WT mice; no such increase occurred in KO mice (Fig. 4c). KO mice showed lower levels of hepatic TG in all diet groups (Fig. 4c), an observation that is in agreement with our biochemical analyses (Table 1). Despite having ≈50% lower total TG content than WT mice, KO mice showed a general trend of increases in the degree of unsaturation in triglycerides in response to EtOH feeding (Fig. 4d). This indicated that, along with an overall decrease in TG synthesis, specific pathways/enzymes involved in polyunsaturated fatty acid (PUFA) synthesis may be stimulated in the livers of KO mice.

### Constitutive activation of liver kinase B1/AMP-activated protein kinase (LKB1/AMPK) pathway in KO livers

**Figure 5:**
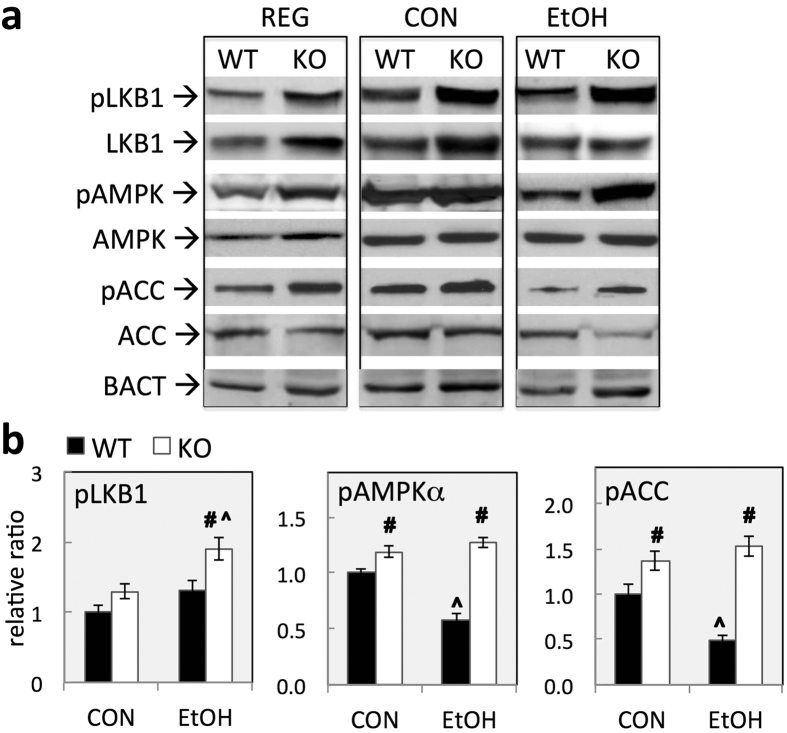
Constitutive activation of LKB1/AMPK pathway in GCLM KO livers. (**a**) Representative Western blotting of key players in LKB1-AMPK pathway in livers from WT and KO mice fed regular chow (REG), control (CON) or ethanol (EtOH) liquid diets for 6 wk. (**b**) Relative levels of phosphorylated proteins. Proteins were quantified by densitometric analysis of protein band intensity. Level of phosphorylated protein was calculated as the ratio to total proteins after normalization to β-actin (BACT). Relative levels are reported as ratios to control (CON-fed WT mice). Data represent mean ± SEM from 4 mice. ^#^*P* < 0.05, vs. diet-matched WT mice. ^^^*P* < 0.0*5*, vs. CON-fed mice of the same genotype.

The resistance of KO mice to steatosis induced by chronic EtOH consumption suggests low hepatic GSH elicits a protective metabolic adaption in the liver. Previous studies suggest that the inhibitory actions of EtOH on the AMPK pathway are crucial for the development of alcoholic steatosis^21^. AMPK, a heterotrimeric kinase composed of catalytic α and regulatory β/γ subunits, is a master regulator of hepatic lipid metabolism^22^. Activation of AMPK by upstream kinases phosphorylates target enzymes, such as acetyl-coA carboxylase (ACC), leading to inhibition of lipogenic pathways and activation of FA oxidation^22^. AMPK also inhibits lipid biosynthesis by suppressing transcription factor sterol regulatory element-binding protein 1 (SREBP1) and promoting the action of peroxisome proliferator-activated receptor alpha (PPARα), a nuclear receptor that acts to enhance both mitochondrial and peroxisomal FA β-oxidation^23^. LKB1, a tumor suppressor, is a major mammalian AMPK kinase in the liver that activates AMPK by phosphorylating the Thr172 residues of AMPKα subunits; importantly, this action appears to be mediated by ROS/RNS^24^. In agreement with observed resistance in KO mice, levels of phosphorylated AMPKα subunit (at Thr172 residue) were higher in the livers of KO mice than in WT mice in all diet groups (Fig. 5). This may have resulted in higher levels of phosphorylation of ACC observed in livers of KO mice (Fig. 5b). Importantly, the observed inhibition of AMPKα phosphorylation by EtOH in WT mice was absent from KO mice (Fig. 5b). In addition, higher levels of phosphorylated (active) LKB1 were noted under these conditions in the livers of KO mice but not WT mice (Fig. 5b). This result is suggestive of the constitutive activation of LKB1/AMPKα pathway in the livers of KO mice. We measured mRNA levels of LKB1 and AMPKα for all diet groups. Our data (supplementary Fig. 2) revealed that expression of these genes were not different between WT and KO livers in any diet groups.

### Differential expression profile of lipid metabolism genes in KO livers following chronic alcohol feeding

**Figure 6:**
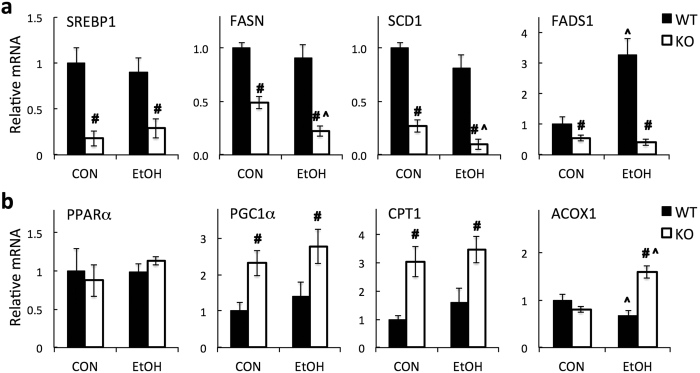
Hepatic gene expression of lipid metabolizing regulators and enzymes. mRNA levels of genes that (**a**) promote lipid synthesis or (**b**) promote fatty acid oxidation were determined by Q-PCR analysis. Relative mRNA abundances are expressed as the fold of control (CON-fed WT mice). Data represent mean ± SEM from 4–6 mice. ^*^P < 0.05, vs. diet-matched WT mice. ^#^*P* < 0.0*5*, vs. CON-fed mice of the same genotype.

Hepatic quantitative real-time PCR analysis revealed that in KO mice fed control chow diet, genes promoting lipid synthesis (*Srebp1*, *Fasn*, *Scd1*, and *Fads1*) were suppressed (Fig. 6a), while genes promoting fatty acid (FA) oxidation (*Pgc-1α* and *Cpt-1*) were induced (Fig. 6b). The combined effect of these changes likely explains the 50% decrease in total hepatic TG content in CON-fed KO mice. When comparing EtOH-fed with CON-fed mice, EtOH feeding did not cause changes in liver mRNA levels of three regulators, namely *Srebp1, Pparα* and *Pgc-1α* in either WT or KO mice (Fig. 6). In WT mice, among the genes examined, *Fads1* (involved in lipogenesis) was found to be up-regulated by EtOH and *Acox1* (catalyzes the first step of peroxisomal FA oxidation) was repressed (Fig. 6). In KO mice, two major lipogenic genes, *Fasn* and *Scd1*, were suppressed by EtOH, whereas *Acox1* was induced (Fig. 6). Notably, *Fasn* and *Scd1* are target genes of SREBP1^25^, and *Cpt-1* and *Acox1* are PPARα/PGC-1α regulated genes^26^. Collectively, the expression profiles of genes involved in lipid metabolism were differentially modulated by EtOH in KO mice (relative to WT mice).

## Discussion

While it is well accepted that EtOH-associated oxidative stress is a key mediator in the pathogenesis of ALD, the molecular details governing this process remain incompletely understood. A causative role of oxidative stress in ALD is supported by several lines of evidence^27^. First, acute *and* chronic EtOH administration induce overproduction of ROS, RNS and other free radicals, observations made in cell cultures, experimental animals and human subjects. Second, EtOH exposure, especially when chronic, reduces the antioxidant capacity of the liver and blood, as reflected by decreased levels of antioxidants (e.g. GSH) and antioxidant enzymes (e.g. superoxide dismutases (SODs), CAT and glutathione peroxidases (GPXs)). Third, pharmacological agents possessing antioxidant properties, such as GSH ester, *N*-acetylcysteine, and vitamins, and antioxidant inducers diminish alcohol-induced liver damage in experimental models. Lastly, genetic manipulation of antioxidant genes, such as *Sod1*, *Gpx*1 and *Cat*, in animal models alters susceptibility to alcohol-induced liver injury^28^,^29^,^30^. Similarly, animals with deficiencies in indirect scavengers of ROS, such as sulfiredoxin and metallothionein, are highly susceptible to alcohol-induced hepatic injury^27^,^31^. In the present study, a mouse model of chronic oxidative stress due to compromised *de novo* GSH synthesis (GCLM KO)^16^ exhibited unexpected protection from chronic alcohol consumption-induced hepatotoxicity and steatosis.

We have previously reported that GCLM KO mice have lower GSH/GSSG and Cys/CySS redox ratios in plasma, resulting in positive thiol reduction potentials, i.e., more oxidized thiol redox states. In the liver, the GSH/GSSG ratio was reduced by two-thirds and redox potential (ΔEGSSG/2GSH) was less negative in KO mice^17^. Collectively, our observations indicate that GCLM KO mice exhibit hepatic and systemic oxidative stress. In the present study, we extend these findings by showing livers from GCLM KO mice exhibit a persistently greater level of oxidant stress as measured by protein carbonylation and expression of redox-sensitive genes. A selective depletion of mitochondrial GSH by chronic ethanol exposure has been consistently reported^32^; this was observed only in WT mice in the present study. Nevertheless, the ethanol-induced deleterious pathological changes in the livers of WT mice are almost completely absent from GCLM KO mice. In line with our observations, BSO co-administration in the final week of a 7-wk ethanol feeding regime in mice, which caused a 72% decline in hepatic GSH, was shown previously to exert a hepatoprotective effect^15^. Thus, through genetic manipulation of GSH biosynthesis, the present study, along with the BSO study, implicates a role for chronic GSH depletion in protection against ethanol-induced hepatotoxicity. Most importantly, this protective phenotype appears to involve the following beneficial cellular adaptions: (i) enhanced metabolism of ethanol and acetaldehyde, (ii) suppression of lipogenic genes and induction of genes involved in fatty acid oxidation, and (iii) induction of the NRF2 antioxidant response and the AMPK metabolic signaling pathway.

Ethanol metabolism plays a significant role in ALD pathogenesis. Its oxidation causes a metabolic shift towards a higher NADH/NAD^+^ ratio that favors enzymatic production of ROS. In addition, acetaldehyde is highly reactive and capable of adducting proteins and lipids that, in turn, initiate a series of deleterious cellular events. Our data suggest that GCLM KO mice have higher capacities for ethanol and acetaldehyde clearance, which may contribute to the protective phenotype. Evaluation on liver enzymes revealed constitutive induction of CYP2E1 and ALDH1A1 in untreated GCLM KO mice. Ethanol-inducible CYP2E1 has an important role in EtOH metabolism, particularly in chronic alcohol users^33^. Rodent ALDH1A1 possesses a very low *Km* (15 μM) for acetaldehyde oxidation^34^, underscoring its significant contribution to acetaldehyde metabolism in this species. Following a 6-wk ethanol feeding, the levels of expression and activity of these enzymes in KO and WT mouse livers were comparable. These results are not consistent with changes in hepatic CYP2E1 or ALDH1A1 mediating enhanced EtOH metabolism in KO mice under the condition of chronic alcohol consumption. Ethanol taken perorally (during feeding) is subject to gastric and hepatic metabolism before reaching the systemic circulation. The decreased blood and liver levels of EtOH and AA in WT in KO mice chronically fed EtOH may relate to delayed gastric emptying and/or increased gastric mucosal of EtOH-metabolizing enzymes. In this context, it would be important to investigate in future studies whether chronic EtOH fed WT and KO mice differ in the gastric metabolism of ethanol. The precise mechanism(s) by which low GSH induces CYP2E1 and ALDH1A1 remain to be elucidated. It should be noted, however, that ALDH1A1, but not CYP2E1, is a downstream target of NRF2, which was found to be activated in untreated GCLM KO mouse liver.

Gene overexpression and knock-down studies have demonstrated a strong association between increased CYP2E1 activity and alcohol-induced fatty liver^35^,^36^. In the present study, low GSH-elicited CYP2E1 induction did not exacerbate liver damage or steatosis following chronic ethanol feeding. Accordingly (and against accepted convention), the contribution of CYP2E1 to ALD pathophysiology may be more complex than originally thought and is likely dependent on the cellular microenvironment or subject to modulation by other signaling pathways^15^,^37^.

After the 6-wk chronic ethanol feeding, the most striking difference between WT and KO mice was the complete prevention of steatosis development in KO mice. A similar protection against steatosis has been previously noted in GCLM KO mice challenged with other liver toxicants^38^ or stress-inducing diets^17^,^39^. In line with this phenotype, liver microarray analyses consistently show dramatic intrinsic suppression of lipogenic genes in GCLM KO mice^17^,^39^. However, no central mechanism for such protection has been reported as yet. In the present study, a panel of genes that promote lipid synthesis was suppressed by 50~80% in naïve (CON-fed) GCLM KO mice. Conversely, a panel of genes that promote fatty acid oxidation was induced 2~3 fold in these same mice. Of these genes, EtOH feeding up-regulated *Fads1* and down-regulated *Acox1* genes in WT mice, a result in agreement with the observed increase in total TG content. However, in GCLM KO mice, genes encoding lipid synthesis enzymes, *Fasn* and *Scd*, were further down-regulated, whereas *Fads1* was up-regulated by ethanol, the net effect of which would be expected to result in lower levels of total TG and increases in the relative abundance of certain lipid species. Liver lipidomics analyses showed that GCLM KO mice displayed a differential change in hepatic lipid profiles in response to chronic ethanol consumption. Specifically, an increase in PUFAs of the TG category was observed in ethanol-fed GCLM KO mice. While ω-3 PUFAs are considered to have beneficial effects^40^, consumption of PUFAs has also been linked with more severe ALD in humans and experimental animals^41^. The biological significance of such a specific increase of PUFA in GCLM KO livers remains to be elucidated. It should be noted that changes in other lipid species that are relevant to liver injury and inflammation, such as acyl-CoAs, diacylglycerides, ceramides and sphingolipids^42^, were not investigated in the present study. More comprehensive lipidomic analyses of the blood and liver from WT and KO mice are currently underway.

Reduced levels of GSH alter the redox state of the cell by decreasing the GSH/GSSG ratio and/or increasing ROS/RNS formation. These changes can cause oxidative modifications to reactive thiols of structurally- or functionally-critical proteins, such as transcription factors^43^, signal transduction molecules^44^ or metabolic enzymes^45^, and thereby evoke cellular defense mechanisms against oxidative damage^46^. Importantly, many of the pathways susceptible to redox modulation are actively involved in the pathogenesis of ALD, including the NRF2 and AMPK pathways. An important protective role of NRF2 against EtOH-induced oxidative stress and lipotoxicity has been established in *in vivo* and *in vitro* studies^47^. The mechanism involves positive regulation of the expression of ALDHs and cytoprotective enzymes (e.g. GCLC, NQO1 and HO-1) and negative regulation on SREBP1 and the inflammatory response^48^. Thus, an increase in NRF2 activity, reflected by induction of NRF2 target genes, likely represent an important mechanism mediating the protection seen in GCLM KO mice. In addition, our results suggest a novel mechanism involving low GSH-associated AMPK activation that prevents alcohol-induced steatosis, which challenges the existing knowledge on the role of low GSH in alcoholic liver disease. One of critical pathogenic events contributing to EtOH-induced lipotoxicity is the inhibitory action of EtOH on the AMPK pathway^21^,^22^. In GCLM KO mouse liver, constitutive activation of AMPKα was observed as measured by levels of phosphorylated AMPKα and phosphorylated ACC; most importantly, higher AMPK activity was maintained after chronic ethanol exposure. This may contribute to the sustained suppression of lipogenic genes (e.g. *Fasn* and *Scd1*) and induction of FA oxidation genes (e.g. *Cpt1* and *Acox1*) in EtOH-treated GCLM KO mouse liver. In addition to maintaining hepatic metabolic homeostasis, AMPK activation has been reported to promote mitochondrial function and suppress endoplasmic reticulum (ER) stress^22^. This effect of AMPK signaling is in agreement with our previous studies showing that, despite having 15% and 40% of WT total and mitochondrial GSH levels (respectively), livers from naïve GCLM KO mice maintain structural and functional integrity of the mitochondria and show induction of ER stress-related genes^17^,^38^. Given that EtOH-induced mitochondrial dysfunction and ER stress are intimately involved in the pathogenesis of ALD, future studies to examine these pathways under the current experimental setting will provide important mechanistic information underlying the protection seen in GCLM KO mice.

Of particular interest in the present study is that AMPK activation appears to be, at least partially, redox-associated. This is based on the observation that the livers of GCLM KO mice in all diet groups have high levels of active (phosphorylated) LKB1, a redox-sensitive kinase and the primary hepatic AMPK kinase^24^. It is possible that the LKB1/AMPK/ACC pathway is activated by direct redox modulation of cellular proteins since activation of AMPK pathway has been directly linked to cellular redox status and hydrogen peroxide^49^. Nonetheless, the role of other upstream signaling pathways cannot be excluded. For example, the silent information regulator 1 (SIRT1), a NAD^+^-dependent protein deacetylase, functions as a key upstream regulator for LKB1/AMPK signaling in the liver^50^. Adipose tissue-derived hormones, such as leptin and adiponectin, are important in modulating hepatic AMPK activity; disruption of these regulators has been documented to contribute to alcohol-induced steatosis^51^,^52^. It should be noted that GCLM KO mice have systemic GSH deficiencies, ranging from 10 to 40% of normal levels depending on the tissue type^16^. As such, it is possible that redox-associated extrahepatic mechanisms may contribute to the activation of the hepatic AMPK pathway.

**Figure 7:**
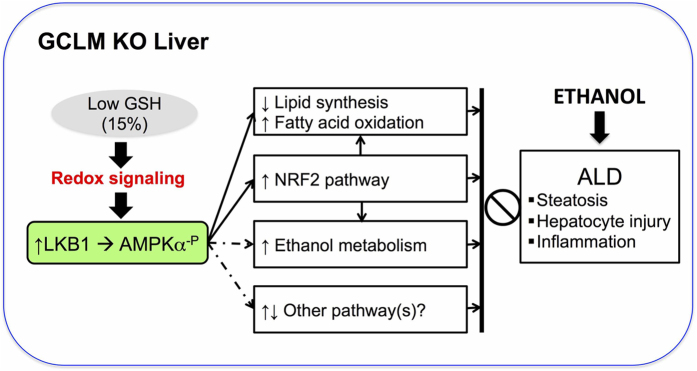
Scheme of proposed mechanisms underlying the protective phenotype of KO mice. We propose that chronic GSH depletion induces redox activation of LKB1/AMPK pathway (through phosphorylation of AMPKα subunit) that serves as a central link triggering multiple metabolic and stress response preventing ALD. These downstream pathways include (enhanced) ethanol metabolism, (suppressed) lipid synthesis and (enhanced) fatty acid oxidation, (activated) NRF2 pathway and unidentified pathways (e.g. ER stress and mitochondrial function).

In conclusion, we show that chronic *in vivo* GSH deficiency, despite inducing CYP2E1 and hepatic oxidative stress, triggers adaptive mechanisms that protect the liver against ethanol-induced toxicity. Protection is likely mediated by redox activation of the AMPK and NRF2 pathways. It should be noted that recent studies have shown convergence between these two pathways such that AMPK activation is an upstream event of the NRF2-mediated antioxidant response^53^. Taken together, we propose that chronic GSH depletion induces redox activation of the AMPK pathway that may serve as the central link triggering mechanisms that prevent alcohol-induced liver injury (as illustrated in Fig. 7). This novel pathway may represent a new therapeutic target for preventing ALD and perhaps non-alcoholic liver steatosis.

## Methods

### Reagents

All chemicals and reagents were purchased from Sigma-Aldrich (St. Louis, MO, USA) unless otherwise specified.

### Animals

The GCLM knockout (KO) mouse line (characterized previously^16^) has been backcrossed into the C57BL/6J background for more than 10 generations. C57BL/6J wild-type (WT) mice were purchased from Jackson Laboratory (Bar Harbor, ME). All animal experiments were approved by and conducted in compliance with Institutional Animal Care and Use Committee (IACUC) of the University of Colorado Anschutz Medical Campus. Mice were maintained in a temperature-controlled room (21–22 °C) on a 12 hr light/dark cycle and supplied with food and water ad libitum.

### Chronic ethanol feeding

Male mice (10–12 wk old) were fed a modified Lieber-DeCarli (LD) diet (F4473SP and F4474SP; Bio-Serv, Frenchtown, NJ) for six wk. The LD liquid diet is composed of 45% fat-derived calories, 15% protein-derived calories and 40% calories comprised varying concentrations of carbohydrate-derived or ethanol (EtOH)-derived calories (EDC). Animals began the study on a diet containing 2% EtOH (v/v) (10.8% EDC) and the amount of EtOH was increased 1% weekly until it reached 5% (v/v) (26.9% EDC) and the animals were maintained on 5% EtOH (v/v) for the remainder of the study. Pair-fed control (CON) mice received a LD diet in which the EtOH content was substituted by carbohydrates. Experimental animals were housed individually and had free access to drinking water. Food intake was recorded daily and body weights were measured weekly. At the end of the 6-wk feeding regimen, mice were euthanized by CO_2_ asphyxiation. Blood was collected by cardiac puncture for biochemical measurements. The liver was quickly removed and weighed. One piece of liver was fixed in 4% paraformaldehyde for liver histology and the remainder frozen in liquid nitrogen for biochemical and gene-protein expression analyses.

### Plasma enzyme assay and liver histology

Plasma was extracted from whole blood and immediately measured for alanine aminotransferase (ALT) and aspartate aminotransferase (AST) activities using a biochemical kit (Diagnostic Chemicals Ltd., Oxford, CT). Liver paraffin sections (5 mm) were prepared and stained with hematoxylin and eosin (H&E) by the Department of Pathology at the University of Colorado Denver using standard procedures. Liver histopathology was examined by a blinded pathologist.

### Pharmacokinetics

Blood EtOH and acetaldehyde pharmacokinetics were determined in male mice (10–12 wk) 0, 1, 3, and 24 hr following an acute administration of EtOH (5 g/kg, i.p.). Mice were euthanized and blood was collected by cardiac puncture and processed immediately for simultaneous determination of EtOH and acetaldehyde by headspace gas chromatography/mass spectrometry (HS-GC/MS) analysis.

### CYP2E1 and ALDH enzymatic activity

CYP2E1 activity was assessed by measuring the rate of oxidation of *p*-nitrophenol (PNP) to *p*-nitrocatechol as described^54^. ALDH activity was determined by monitoring the formation of NADH at 340 nm during the oxidation of propionaldehyde as described^55^.

### HS-GC/MS analyses

Blood and tissue concentrations of EtOH and acetaldehyde from pharmacokinetics study and at the end of 6-wk ethanol feeding regimen were determined by HS-GC/MS as described^56^. Briefly, whole blood (≈600 μl) was immediately mixed with 2x volume of ice-cold 1.7 M perchloric acid solution to prevent the spontaneous formation of acetaldehyde. Frozen livers (≈100 mg) were homogenized in 8x volume of ice-cold 0.6 M perchloric acid solution. Proteins were removed by centrifugation at 12,000 rpm at 4 °C for 20 min and the supernatant was transferred to a glass vial and sealed tightly. HS-GC/MS analysis was performed using a GCMS-QP2010 system (Shimadzu, Kyoto, Japan) with an AOC-5000 auto-injector (Shimadzu, Kyoto, Japan) with an AQUATIC capillary column (internal diameter 60 m × 0.25 mm; film thickness 1.0 μm) (GL Sciences, Tokyo). Peaks were identified in selected ion monitoring method as follows: acetaldehyde by 28.95 m/z with reference ion; 43.00 and 44.00 m/z, and EtOH by 31.00 m/z with reference ion; 29.30 and 45.00 m/z. The concentration of EtOH and acetaldehyde were calculated according to respective standard curves.

### Subcellular GSH levels

Liver mitochondrial and cytosolic fractions were prepared from frozen liver samples as described^17^. GSH levels were determined spectrophotofluorometrically in these fractions as described^57^.

### Analysis of liver cholesterol ester (CE) and triglycerides (TG)

Total liver TG content was determined biochemically from frozen liver pieces^58^ using the triglyceride reagents (Sigma, St. Louis, MO). Lipidomic profiling of CE and TG species was performed by mass spectrometry-based analysis. Analysis of CE molecular species was performed as previously described^59^. Briefly, frozen livers were allowed to thaw to room temperature and homogenized by macerating with a disposable glass pipet. Liver sample (10 mg) was then mixed with K_2_SO_4_ (1 g) and a single reference standard of CE (18:0) (250 ng), followed by two extractions with 2 ml of 1:1 methyl tert-butyl ether/hexane. The organic layers from two extractions were combined and aliquots were analyzed on a 4000 Q-Trap mass spectrometer (AB SCIEX, Framingham, MA) using the 50 × 2.1 mm Hydrophilic Interaction Liquid Chromatography (HILIC-LC) column (Waters Corp., Milford, MA). Individual molecular species of CE were analyzed using multiple reaction monitoring transitions and quantified according to the reference standard and reported as μmol/g liver tissue. Total liver CE content was calculated by summing individual molecular species. For TG analysis, total liver samples were extracted with chilled water-methanol-chloroform mixture (3:4:8) containing LPC (19:0) and chlorpropamide as internal standards. The organic layer was collected and evaporated to dryness under nitrogen flow. Lipid extracts were reconstituted in 50% methanol and analyzed using a UPLC-ESI-QTOF platform. Acquity UPLC CSH™ C18 (1.7 μm, 2.1 × 100 mm) column (Waters Corp.) was used to separate constituent lipid species. The mobile phase comprised of (A) 10 mM ammonium formate in 60% aqueous acetonitrile containing 0.1% formic acid and (B) 10 mM ammonium formate in 9:1 mixture of isopropanol and acetonitrile with a 0.5 mL/min flow rate maintained during a 20 min run. The QTOF SYNAPT HDMS mass spectrometer was operated in both electrospray ionization positive (ESI+) and negative ESI (ESI−) for analysis of lipid composition. Sulfadimethoxine was used as the lock mass (m/z 311.0814+) for accurate mass calibration in real time. For MS/MS fragmentation of target ions, collision energy ranging from 10 to 50 eV was applied with argon as the collision gas. Samples were analyzed in a randomized fashion. Lipid species were identified using accurate mass and fragmentation pattern. The relative abundance of lipids were determined from normalized response with respect to internal standards, calculated using area under the curve of the extracted chromatogram for individual lipids. Total liver content was calculated by summing individual molecular species. The fold change in the abundance of each lipid species in WT or KO mice following EtOH treatment were normalized relative to abundance in respective control groups.

### Western immunoblot analyses

For the preparation of total liver homogenates, frozen liver pieces were homogenized in RIPA buffer (150 mM NaCl, 1% TritonX-100, 0.25% sodium deoxycholate, 0.1% SDS, 50 mM Tris, 1 mM EDTA, 1 mM PMSF, protease inhibitor cocktail, pH 7.4) on ice with a tissue tearor (BioSpec Products, Bartlesville). Tissue homogenates were centrifuged at 12,000 rpm at 4 °C for 20 min and the supernatant was collected. For the preparation of nuclear extracts, frozen liver pieces were homogenized in homogenization buffer (20 mM HEPES, 70 mM sucrose, 220 mM Mannitol, 2 mM EDTA, 0.5 mg/ml BSA, 0.1 mM PMSF, 1.0 mM DTT, pH 7.5). The homogenates were centrifuged at 500 × g for 15 min; resultant supernatant fractions were centrifuged at 1,000 × g for another 15 min, followed by centrifugation of the supernatant at 3,000 × g for 15 min. The pellets were resuspended in RIPA buffer and centrifuged at 14,000 × g to acquire nuclear extracts. Proteins in total homogenates (20–40 μg) or nuclear extracts (40 μg) were resolved by 10% SDS-PAGE and immunoblotted using following primary antibodies: chicken polyclonal antibodies against mouse GCLM^7^ and mouse ALDH1A1^60^; rabbit polyclonal antibodies against human ADH1, catalase, NRF2, CYP2E1 (all from Santa Cruz, Dallas, Texas), ALDH1B1^61^, and ALDH2^62^; rabbit monoclonal antibodies against total and phosphorylated AMPKα, AMPKβ, ACC and LKB1 (all from Cell Signaling, Danvers, MA); mouse monoclonal antibodies against β-actin and lamin-A (Sigma, St. Louis, MO). Corresponding horseradish peroxidase-conjugated secondary antibodies were purchased from Sigma (St. Louis, MO) and used at 1:5,000 according to the manufacturer’s protocol. Chemiluminescence was visualized on a scanner (Storm 860, Molecular Dynamics, Sunnyvale, CA) or using film. Protein band intensity was quantified using the ImageJ program (rsbweb.nih.gov/ij/download.html) and normalized with β-actin (for total homogenate) or lamin-A (for nuclear extracts). Results are reported as fold of control as specified.

### Reverse transcription and real-time quantitative PCR (Q-PCR)

Total RNA was isolated from frozen liver pieces using Tri-Reagent (Molecular Research Center, Inc.) according to manufacturer’s protocol. cDNA was synthesized using Superscript III First-Strand Synthesis System (Invitrogen, Carlsbad, CA) according to manufacturer’s instructions using 1 μg total RNA in a 20 μl reaction volume. Q-PCR reaction mixtures contained 1 μl cDNA, 1x SYBR Green Supermix (BioRad, Hercules, CA), and 0.15 μM gene-specific primer sets in a total volume of 25 μl. Sequences of primers used for Q-PCR can be found as Supplementary Table 1. Reactions were run using the DNA Engine Opticon 2 Continuous Fluorescence Detector System (Applied Biosystem, Grand Island, NY). Expression of β-2-microtubilin (B2M) was used for normalization of CT data according to the ΔΔCT method^63^. Relative mRNA levels of individual genes were reported as fold value of the control.

### Detection of protein carbonylation

Levels of carbonylated proteins were determined with the OxyBlot protein oxidation detection kit following the manufacturer’s protocol (Millipore, Billerica, MA, USA). Visualization, protein quantification and result expression were identical as protein detection in tissue homogenates (above).

### Statistical analyses

Statistics were performed using SigmaStat Statistical Analysis software (SPSS Inc., Chicago, IL). Group means were compared by one-way ANOVA, followed by a Student’s unpaired *t*-test. Results are reported as the mean ± SEM. *P* < 0.05 was considered significant.

## Additional Information

**How to cite this article**: Chen, Y. *et al.* Chronic Glutathione Depletion Confers Protection against Alcohol-induced Steatosis: Implication for Redox Activation of AMP-activated Protein Kinase Pathway. *Sci. Rep.* **6**, 29743; doi: 10.1038/srep29743 (2016).

## Supplementary Material

Supplementary Information

## Figures and Tables

**Table 1:** Caloric intake, body weight, liver weight, serum liver enzyme activities and hepatic triglyceride content.

Parameter	CON		EtOH	
	WT	KO	WT	KO
Daily intake (calories)	16.2 ± 0.2	16.2 ± 0.5	16.6 ± 0.3	17.0 ± 0.8
Body weight gain (g)	7.7 ± 1.1	7.0 ± 0.7	1.5 ± 0.4^a^	4.0 ± 0.4^a,b^
Liver weight (% body weight)	4.1 ± 0.2	4.6 ± 0.1	5.1 ± 0.1^a^	4.6 ± 0.1^b^
Serum ALT (U/dL)	18.8 ± 4.1	23.0 ± 2.4	39.8 ± 3.3^a^	26.3 ± 4.9^b^
Serum AST (U/dL)	45 ± 19	32 ± 17	64 ± 15	44 ± 11
Hepatic triglyceride content (mg/g liver)	39.3 ± 2.3	18.5 ± 2.4^c^	61.0 ± 4.8^a^	23.8 ± 2.7^b^

Mice were fed EtOH containing or isocaloric control (CON) liquid diet for 6 wk. Food intake was recorded daily and body weight was measured weekly. Other parameters were examined at the end of the 6 wk feeding regimen. Data are presented as mean ± SEM from 6 mice. ^a^*P < 0.05*, Student’s unpaired t-test with *post-hoc* Bonferroni correction, vs. CON-fed mice of the same genotype. ^b^*P < 0.05*, Student’s unpaired t-test with *post-hoc* Bonferroni correction, vs. EtOH-fed WT mice. ^c^*P* < 0.05, Student’s unpaired t-test with *post-hoc* Bonferroni correction, vs. CON-fed WT mice.
