# Supplementary material for: Chronic Glutathione Depletion Confers Protection against Alcohol-induced Steatosis: Implication for Redox Activation of AMP-activated Protein Kinase Pathway
Source: Sci Rep. 2016 Jul 12;6:29743. doi: 10.1038/srep29743 (PMC4940737; doi:10.1038/srep29743)
Supplement: Supplementary Information [file srep29743-s1.doc]

**Chronic Glutathione Depletion Confers Protection against Alcohol-induced Steatosis: Implication for Redox Activation of AMP-activated Protein Kinase Pathway**

**Ying Chen^1†^, Surendra Singh^1†^, Akiko Matsumoto^2^, Soumen K. Manna^3^, Mohamed A. Abdelmegeed^4^, Srujana Golla^3^, Robert C. Murphy^5^, Hongbin Dong^1^, Byoung-Joon Song^4^, Frank J. Gonzalez^3^, David C. Thompson^6^, and Vasilis Vasiliou^1*^**

^1^Department of Environmental Health Sciences, Yale University, New Haven, CT 06250

^2^Department of Social Medicine, Saga University School of Medicine, Saga, 849-8501 Japan

^3^Laboratory of Metabolism, Center for Cancer Research, National Cancer Institute, Bethesda, Maryland 20852

^4^Laboratory of Membrane Biochemistry and Biophysics, National Institute on Alcohol Abuse and Alcoholism, National Institutes of Health, Bethesda, MD 20892

^5^Department of Pharmacology, University of Colorado AMC, Aurora, CO 80045

^6^Department of Clinical Pharmacy, University of Colorado AMC, Aurora, CO 80045.

*Corresponding author: Vasilis Vasiliou, PhD, Department of Environmental Health Sciences, Yale School of Public Health, 60 College St, New Haven, CT 06250, Tel: 203 737 8094; Fax: 203 724 6023; Email: [vasilis.vasiliou@yale.edu](mailto:vasilis.vasiliou@yale.edu).

^†^Authors contributed equally.

**Supplementary data**

**Supplementary Table 1. Primer sets used in QPCR analysis**

| **Gene** | **Primer sequence** |
| --- | --- |
| Glutamate cysteine ligase catalytic subunit (*Gclc*) | F: 5’-CCCAGACTAGGCTGTCCT-3’  R: 5’GGTGTATTCTTGTCCTTGAATATTGGC-3’ |
| Metallothionein I (*Mt1*) | F: 5'-CACGACTTCAACGTCC-3’  R: 5'-CAGCAGGAGCAGCAGCTCTTCTTGCAG-3’ |
| Heme-oxgenase 1 (*Hmox1)* | F: 5’-GCCACCAAGGAGGTACACAT-3’  R: 5’-GCTTGTTGCGCTCTATCTCC-3’ |
| Liver kinase B1 (*Lkb1*) | F: 5’-GCCTCCTGAGATTGCCAATG-3’  R: 5’-GGTACAGGCCCGTGGTGAT-3’ |
| AMP-activated protein kinase alpha-1 subunit (*Ampkα1*) | F: 5’-AAGCCGACCCAATGACATCA-3’  R: 5’-CTTCCTTCGTACACGCAAAT-3’ |
| Sterol regulatory element-binding protein 1 *(Srebp1*) | F: 5′-CATCGAAGACATGCTCCAG-3’  R: 5′-TGTCACCTTGGGTCCTC-3’ |
| Peroxisome proliferator-activated receptor α (*Ppara)* | F: 5’AGGCAGATGACCTGGAAAGTC-3’  R: 5’ATGCGTGAACTCCGTAGTGG-3’ |
| PPAR-γ coactivator 1α (*Pgc-1α*) | F: 5’-TATGGAGTGACATAGAGTGTGCT-3’  R: 5’-CCACTTCAATCCACCCAGAAAG-3’ |
| Fatty acid synthase (*Fasn*) | F: 5’-AGACAGAGAAGAGCCATGGAGGA-3’  R: 5’-ATCCTTCAGCTTTCCAGACCGCTT-3’ |
| Stearoyl-CoA desaturase 1 (*Scd1*) | F: 5’-TCTACACCTGCCTCTTCGGGATTT-3’  R: 5’-TTCGTACACGTCATTCTGGAACGC-3’ |
| Fatty acid elongase 6 (*Elovl6*) | F: 5’-GCAGTTCAACGAGAACGAAGCCAT-3’  R: 5’-GCACCGAATATACTGAAGACGGCA-3’ |
| Fatty acid desaturase 1 (*Fads1*) | F: 5’-CATGGAATCACCTGCTA AT-3’  R: 5’-GGGTCCGATGAGGA GA-3’ |
| Fatty acid desaturase 2 (*Fads2)* | F: 5’-CGTGTCATCGGACACTATTC-3’  R: 5’-TCGGTGATCTGAGAGCTT-3’ |
| Fatty acid elongase 2 (*Elovl2)* | F: 5’-CCATGGAGCAGCTGAAG-3’  R: 5’-ACTTGTTACCCAGCCATATC-3’ |
| Fatty acid elongase 5 (*Elovl5*) | F: 5’-GCTCAGGCAGAGAGGTTT-3’  R: 5’-CTGCCGGTTCTTCATGTATTT-3’ |
| Carnitine palmitoyltransferase 1 (*Cpt-1*) | F: 5’-GGACGAATCGGAACAGGGATA-3’  R: 5’-CCTTGTAATGTGCGAGCTGCA-3’ |
| Acyl-CoA oxidase 1 (*Acox1*) | F: 5’-CCGCCACCTTCAATCCAGAGTTA-3’  R: 5’-TCACAGTTGGGCTGTTGAGAATG-3’ |
| Cytochrome P450 4A10 (*Cyp4a10*) | F: 5’-GCTCCTTGGATTGGGTATG-3’  R: 5’-GACAGTGTCTAGGGTCATTAAG-3’ |
| Cytochrome P450 4A14 (*Cyp4a14*) | F: 5’-CCATACCCAAAGGTATCACAG-3’  R: 5’-CACCTTCAGCTCGTTCATAG-3’ |
| β-2 microglobulin **(***B2m*) | F: 5’-CATGGCTCGCTCGGTGACC-3’  R: 5’-AATGTGAGGCGGGTGGAACTG-3’ |

Forward (F) and reverse (R) primer sequences are presented for each gene-specific QPCR reaction.

**
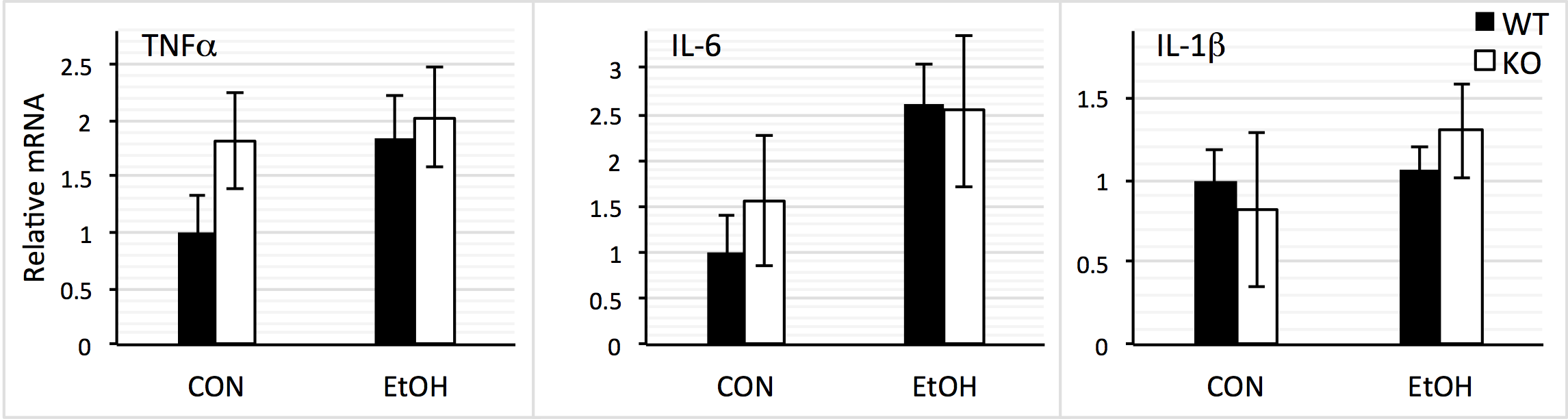
**

**Supplementary Fig. 1. Hepatic expression of inflammatory genes.** mRNA levels of tumor necrosis factor α (TNFα), interleukin 6 (IL-6) and interleukin 1β (IL-1β) were determined by Q-PCR analysis. Relative mRNA abundances are expressed as the fold of control (CON-fed WT mice). Data represent mean ± SEM from 4 mice. Group means were compared by one-way ANOVA, followed by a Student’s unpaired *t*-test. No significant differences were observed between WT and KO mice in any diet groups. CON = control liquid diet; EtOH = ethanol-containing liquid diet.

**
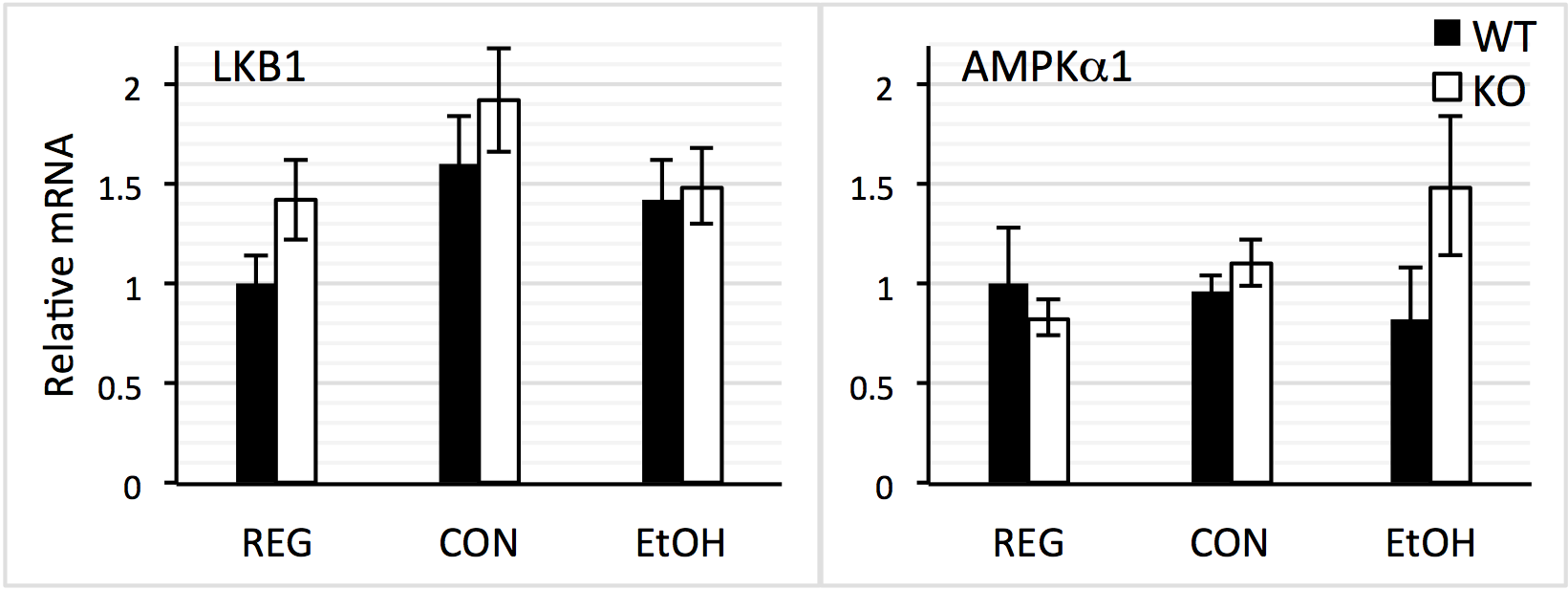
**

**Supplementary Fig. 2. Hepatic gene expression of LKB1 and AMPKα1.** mRNA levels of genes were determined by Q-PCR analysis. Relative mRNA abundances are expressed as the fold of control (REG-fed WT mice). Data represent mean ± SEM from 4 mice. Group means were compared by one-way ANOVA, followed by a Student’s unpaired *t*-test. No significant differences were observed between WT and KO mice in any diet groups. REG = regular chow; CON = control liquid diet; EtOH = ethanol-containing liquid diet.
